# Capability of Tissue Stem Cells to Organize into Salivary Rudiments

**DOI:** 10.1155/2012/502136

**Published:** 2012-03-15

**Authors:** Kenji Okumura, Masanori Shinohara, Fumio Endo

**Affiliations:** ^1^Department of Pediatrics, Kumamoto University School of Medicine, Honjo 1-1-1, Kumamoto 860-8556, Japan; ^2^Department of Oral and Maxillofacial Surgery, Kumamoto University, Kumamoto, Japan

## Abstract

Branching morphogenesis (BrM), an essential step for salivary gland development, requires epithelial-mesenchymal interactions. BrM is impaired when the surrounding mesenchyme is detached from the salivary epithelium during the pseudoglandular stage. It is believed that the salivary mesenchyme is indispensable for BrM, however, an extracellular matrix gel with exogenous EGF can be used as a substitute for the mesenchyme during BrM in the developing salivary epithelium. Stem/progenitor cells isolated from salivary glands in humans and rodents can be classified as mesenchymal stem cell-like, bone-marrow-derived, duct cell-like, and embryonic epithelium-like cells. Salivary-gland-derived progenitor (SGP) cells isolated from duct-ligated rats, mice, and swine submandibular glands share similar characteristics, including intracellular laminin and *α*6*β*1-integrin expression, similar to the embryonic salivary epithelia during the pseudoglandular stage. Progenitor cells also isolated from human salivary glands (human SGP cells) having the same characteristics differentiate into hepatocyte-like cells when transplanted into the liver. Similar to the dissociated embryonic salivary epithelium, human SGP cells aggregate to self-organize into branching organ-like structures on Matrigel plus exogenous EGF. These results suggest the possibility that tissue stem cells organize rudiment-like structures, and the embryonic cells that organize into whole tissues during development are preserved even in adult tissues.

## 1. Introduction

 Salivary glands are small digestive organs that have a wide variety of functions and vary greatly in the dominant cell type in acini as well as cytodifferentiation of the acinar cells depending on the major glands. Salivary glands synthesize and secrete a large variety of polypeptides including growth factors that have systemic effects. Both epidermal growth factor (EGF) and nerve growth factor (NGF) isolation from mouse salivary gland are especially well known since researchers studying the topic were awarded a Nobel prize [1]. The EGF system regulates not only gastrointestinal mucosal constancy in adults, but salivary gland development during embryonic periods.

 In this paper, we first discuss the histological and developmental biological aspects of the salivary gland, which are helpful for understanding their stem/progenitor cell characteristics. Branching morphogenesis (BrM) is a developmental process for epithelial cell-forming branching tubules that are present in various exocrine organs such as the lungs as well as mammary, prostate, and lacrimal glands. BrM is well characterized in the salivary glands, and these glands have contributed as a good experimental model in developmental biology for over 50 years. BrM is a result of epithelial-mesenchymal interactions and is regulated by extracellular matrix (ECM) composition and growth factors. Many ECM proteins including collagens, laminins, proteoglycans, and fibronectin play important roles in salivary gland morphogenesis [2]. Among these proteins, laminins are essential components of the basement membrane (BM), and ECM receptor integrins expressed on salivary glands epithelia play important roles in BrM. Perturbations in laminins and integrin interactions induce abnormal BrM. Interestingly, the BrM process does not necessarily demand mesenchymal cells, and BM-like substratum plus exogenous EGF induce BrM in mouse salivary rudiment *in vitro *[3]. We also discuss the EGF system in both BrM and epithelial-specific cytodifferentiation.

 Next, we introduce experimental regeneration models developed by using methods such as irradiation and duct ligation, which are used to understand the histopathological reactions of salivary gland tissues. We also discuss the evaluation of the characteristics of representative stem/progenitor cells isolated from adult salivary glands such as mesenchymal stem cell-like cells (SGSCs), bone-marrow-derived cells (BMDCs), c-kit-positive excretory duct cells, intercalated duct (ID) cells, and salivary gland-derived progenitor (SGP) cells. Among these candidates, SGP cells are involved in intracellular laminin production and *α*6*β*1-integrin expression. Finally, we show how human SGP cells could self-organize to constitute rudiment-like structures in culture only with Matrigel plus exogenous EGF. This result suggests the possibility that tissue stem cells are capable of organizing rudiment-like structures.

## 2. Histology and Organogenesis of the Salivary Glands

### 2.1. Histology

 Salivary glands are classified into 2 types: major and minor salivary glands. There are 3 major glands: the parotid, submandibular (SMG), and sublingual glands. Each of these 3 major salivary glands has different tissue architecture and a dominant acinar cell type. There are 2 types of acinar cells: serous- and mucous-type acinar cells; the serous/mucous distinction is different among the major salivary glands.  Table 1 lists the acinar cell types of the major salivary glands in humans [4, 5], mice [6], and rats [6–9].

 The serous/mucous distinction in developing salivary glands changes even after birth. Parotid glands are purely serous in adult rats, whereas mucous-type acinar cells are present in neonate rat parotid glands (days 1–8) [7]. In contrast, the sublingual glands of adult rats are mainly mucous. However, periodic-acid-Schiff-(PAS-) positive serous-type cells are more numerous than Alcian blue-positive mucous-type cells in E19.5 rat sublingual glands [4]. These observations indicate that the dominant acinar cell type in developing parotid and sublingual glands is converted from the late prenatal to the early postnatal period. In neonate sublingual glands, acinar cells have numerous serous granules that are replaced by mucous granules according to the developmental stage [8]. The lack of acinar cell apoptosis in these glands indicates that most of the acinar cells transform, but they are not replaced by cells of another type. In the case of rat SMG, there are 2 distinct types of secretory cells in the maturing acini: terminal tubule cells (Type I) and proacinar cells (Type III) that are discriminated on the secreting proteins: protein C or B1-immunoreactive proteins. During development, terminal tubule cells disappear and proacinar cells differentiate into mature seromucous acinar cells during the first 3 postpartum weeks [10]. It is unclear why the major salivary glands vary greatly in the dominant cell type and cytodifferentiation sequence in acinar cells.

 From acini to the excretory ducts, saliva flows through 2 types of ducts: intercalated and striated ducts. The ID connecting the acinar portion and striated duct is lined with flat, spindle-shaped duct epithelial cells. The characteristics of ID epithelial cells are discussed below. Striated ducts consist of eosinophilic columnar cells with basal striations; these striations exhibit infoldings of the plasma membrane at the basal site of the duct cell. The numerous mitochondria in this region generate the energy required to drive sodium resorption and potassium secretion from saliva. After puberty, granular convoluted tubules (GCTs) are found between the intercalated and striated ducts. Although GCT cells are the major site of growth-factor synthesis in the salivary glands of mice and rats, human salivary glands lack GCT cells; in humans, growth factors are usually synthesized in striated duct cells. Finally, saliva-containing digestive enzymes, antimicrobial substances, mucins, and growth factors flow to the oral cavity and moves to the downstream gastrointestinal tract [11].

### 2.2. Role of EGF Secreted by Salivary Glands

 Although the salivary glands are components of the digestive system and amylase secretion is their best-known excretory function, the salivary glands secrete many other substances into the oral cavity [12]. For example, saliva includes the following growth factors: epidermal growth factor (EGF), fibroblast growth factor (FGF), nerve growth factor (NGF), insulin and insulin-like growth factor family proteins (IGF-I and IGF-II), transforming growth factor-*β* (TGF-*β*) family members, and transforming growth factor-*α* (TGF-*α*). These growth factors are synthesized and secreted by the ductular cells of the granular convoluted tubules [13, 14].

 Saliva contributes to the protection and repair of the oral mucosa, which frequently sustains injuries from the external environment. Wounds in the oral mucosa heal faster than cutaneous wounds; for example, an ulcer in the oral mucosa caused by the teeth is repaired faster than a finger wound. Sialoadenectomized animals exhibit gastric ulcers in the intestinal mucosa [15], delayed oral wound healing [16], and delayed liver regeneration after partial hepatectomy [17]. These gastrointestinal tract phenomena result from systemic EGF shortages. However, the salivary glands are not the only tissues that synthesize EGF. Brunner's glands in the duodenum secrete EGF, which flows to the liver via the portal vein and contributes to hepatic regeneration but is not sufficient for complete regeneration. Mouse salivary gland produces 80% of the EGF protein present in mice; the EGF protein was first isolated from the salivary glands of male mice in 1962 [1].

 The main function of EGF is to regulate epithelial growth and proliferation; therefore, EGF contributes to the maintenance of the gastrointestinal tract epithelia in adult animals. Moreover, EGF plays an important role in salivary gland organogenesis, especially in BrM. During salivary gland development (E13–18), mRNA transcripts for EGF pathway molecules, including EGF, TNF-*α*, and EGF-R, are present in rudiments [18]. The pre-EGF mRNA transcription level reaches a significant peak at E16 [18], and EGF protein is present in both the terminal bud and stalk during the embryonic period.

### 2.3. Branching Morphogenesis

**Figure 1:**
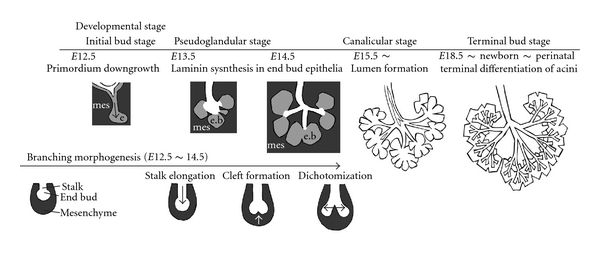
Brief overview of salivary gland development in mice. Schematic diagram of the 4 stages of salivary gland development: initial bud, pseudoglandular, canalicular, and terminal bud stage. Laminin-producing epithelia in end buds are indicated in light grey (pseudoglandular stage). Laminin-producing epithelia are only present in end buds but not in the stalk portion during BrM. A schematic diagram of the 3 steps in branching morphogenesis, stalk elongation, cleft formation, and dichotomization is also shown. Salivary mesenchyme is indicated in dark grey, and epithelia in white. See text for explanation. Abbreviations: mes: mesenchyme; e: epithelium; e.b: epithelial bud.

 All exocrine glands and some other organs with branched morphology, such as the lacrimal glands, salivary glands, mammary glands, lungs, liver, pancreas, and kidneys, develop via a basic developmental program called BrM. BrM is a repetitive process consisting of the following 3 steps: (1) stalk elongation, in which the epithelial cluster surrounding the mesenchyme elongates to form a stalk portion; (2) cleft formation, in which a cleft is indented at the sharp end of the epithelial cluster; (3) dichotomization, in which a cleft cleaves the epithelial cluster into 2 parts. Each segmentized cluster elongates to form each stalk. The epithelial clusters undergo the BrM process again to form highly branched acinotubular structures (Figure 1).

 Salivary gland morphogenesis occurs in 4 stages: the initial bud, pseudoglandular, canalicular, and terminal bud stages [6]. The oral epithelium on the floor of the oral cavity begins its downward growth into the underlying mesenchyme at E11.5, which marks the beginning of salivary gland development. The following day, the BrM begins to form a deep cleft at the epithelial bud, generating 3–5 epithelial endopieces surrounded by a capsule of condensed mesenchyme. An early network of an epithelial branch with a terminal bud is formed by E13.5. The epithelial clusters at the end of each stalk are called “terminal buds.” BrM proceeds until the late pseudoglandular stage (E14.5). The majority of ducts develop a lumen between the canalicular stage (E15.5) and terminal bud stage (E18.5), in which proacinar maturation begins and immunolocalization of the mucin protein in acinar cells is detected. In summary, BrM is mainly observed from E12.5–14.5 (Figure 1).

### 2.4. Mesenchyme-Dependent Morphogenesis

 BrM extension depends on epithelial-mesenchymal interactions; the lack of factors from either results in developmental failure. Separation of the mesenchyme from the epithelial bud at E13 after trypsinization results in failed epithelial branching *in vitro* [19]. However, the epithelial buds undergo BrM again if they are brought in contact with mesenchyme. Abnormal BrM is observed when the epithelial bud is separated from the mesenchyme by a thin filter [19]. These findings indicate that epithelial buds require both soluble substances secreted by the mesenchyme and direct contact with the mesenchyme itself for BrM.

 The mesenchymal tissues vary across regions in the developing body. Heterotypic recombination of the epithelium and mesenchyme indicates that only salivary mesenchyme induces BrM in salivary gland epithelium from the initial bud stage. Mammary mesenchyme cannot induce BrM in the salivary epithelium. However, the mammary epithelium assumes a glandular structure similar to that of the salivary glands when recombined with the salivary mesenchyme [20]. Mammary epithelia recombined with salivary mesenchyme synthesizes milk protein and *α*-lactalbumin [21]. In short, morphogenesis (i.e., tissue architecture) depends on the type of mesenchyme, and the cytodifferentiation aspect of the epithelium is predetermined by the developmental background including its origin. However, early epithelium is more flexible than later epithelium. For example, the pituitary epithelium of E9–11 mice differentiates into *α*-amylase-positive acinar cells when recombined with SMG mesenchyme [22]. Therefore, epithelial cytodifferentiation depends on the stage of development.

 It has long been believed that the mesenchyme is essential for epithelial morphogenesis (i.e., mesenchymal requirement). However, the combination of basement membrane-like substratum (Matrigel) and exogenous EGF/TGF-*α* could be a fitting substitute for the salivary mesenchyme since the salivary epithelium undergoes typical BrM with this combination [3]. This study indicates that mesenchymal cells are not required for BrM, at least in salivary gland development. The indispensable components of BrM form a direct contact between the extracellular matrix (ECM) and epithelium and lead to activation of the EGF system in the salivary epithelium. EGF is synthesized in the epithelium during the canalicular/terminal bud stage; however, it is not evident whether salivary mesenchymal cells secrete EGF. However, mouse embryonic palatal mesenchymal cells produce EGF/TGF-*α* in the developing oral cavity and regulate the production of various types of ECMs [23]. EGF is not expressed in mouse SMG mesenchyme during morphogenesis, specifically the initial bud to terminal bud stage [24]. However, neuregulin1, an EGF family ligand expressed in E13-mesenchymes, plays an essential role in BrM [25].

### 2.5. Disruption of Laminin and Integrin Interaction Perturbs BrM

 Direct contact between ECM proteins and the expanding epithelium is essential for BrM in developing organs. The BM bordering the epithelium and surrounding the mesenchyme is a thin sheet-like structure assembled by ECM proteins. The laminin family of glycoproteins is a major constituent of both the BM and Matrigel that could be a substitute for mesenchyme in BrM, as described above. Sixteen different heterotrimers have been identified, and each laminin is assembled from *α*, *β*, and *γ* subunits [26]. Laminin isoforms have distinct temporal distributions in the developing mouse SMG. For example, the laminin-*α*1 and laminin-*α*5 subunits are expressed at the terminal bud, while the laminins *α*1, *α*3, and *α*5 subunits are expressed at the stalk in E13 mice SMG. Laminin plays an important role in development, and mice lacking laminin *α*1, *β*1, or *γ*1 are embryonic lethal. However, laminins *α*2, *α*3, and *α*4 chain-disrupted mice do not exhibit severe abnormalities and have normal BrM. The most important laminins in development are laminin-1 (*α*1*β*1*γ*1) and laminin-10 (*α*5*β*1*γ*1).

 Integrins, a large family of transmembrane proteins composed of *α*- and *β*-integrin subunits, are the laminin receptors expressed on the epithelium. Eighteen *α*- and 8 *β*-subunits are assembled into 24 heterodimers; 6 integrins—*α*1*β*1, *α*3*β*1, *α*6*β*1, *α*7*β*1, *α*2*β*2, and *α*6*β*4—bind to laminin [26]. The use of blocking antibodies in SMG organ culture is useful for elucidating the functions of laminins and integrins in SMG development. Antibodies against the *α*6-integrin subunit [18], laminin-*α*1 [27], and the nidogen-binding domain on laminin-*γ*1 [28] perturb BrM and cause severe terminal bud number reduction. Impaired morphogenesis with less branching and scant lobulated terminal buds indicates that laminin and integrin interaction is important for cleft formation in BrM.

 Underlying mesenchyme cells synthesize and secrete laminins, and the epithelium expresses the laminin-receptor *α*6-integrin subunit or dystroglycan. Surprisingly, laminin and collagen IV synthesis is activated specifically in the epithelium of the distal end of each branch on days 15–17 of gestation in rats (pseudoglandular stage: E13-14 in mice) [29]. Laminin is not detected in the stalk portion of the epithelium. Terminal bud epithelia synthesize laminin for a specific period (E15–19 in rats), indicating that BM components are synthesized by both surrounding mesenchymal cells and epithelial cells. Salivary epithelia are stimulated to synthesize ECM proteins to compensate for the rapid enlargement of the BM. These findings indicate that the rapidly growing embryonic SMG epithelium simultaneously synthesizes laminin protein and expresses its receptor, the *α*6-integrin subunit. Expression of *α*6-integrin in mouse SMG epithelium is regulated by the EGF system [18].

### 2.6. Epithelial-Specific Cytodifferentiation

 The EGF system is important for BrM and epithelial maturation in salivary glands. Synthesis of the *α*6-integrin subunit in SMG epithelium is activated by EGF and is drastically reduced by EGF-R tyrosine kinase inhibitors. The EGF system plays a physiological role in BrM by regulating expression of the *α*6-integrin subunit.

 EGF-R-deficient mice survive only 8 days after birth, and they have impaired epithelial development in organs such as the lungs and the gastrointestinal tract [30]. BrM was impaired and the epithelial branch number was significantly reduced in the submandibular glands of EGF-R-deficient mice. In addition, inactivation of EGF-R led to mesenchymal cell apoptosis adjacent to the end-bud where EGF-R-expressing cells were located [31]. EGF-R is constitutively expressed in the epithelium of the normally developing salivary gland, but acinar cell cytodifferentiation in EGF-R-deficient mice was not histologically abnormal [24]. However, the EGF-R inhibitor gefitinib impaired epithelial maturation of the *in vitro* cultured E13 salivary rudiment [31]. The EGF system is more critical for morphogenesis than for epithelial maturation and cytodifferentiation.

 Hypohydrotic ectodermal dysplasia (HED) is an inherited disease caused by mutation of ectodysplasin-A (*EDA*). The mouse homologue of *EDA* is tabby (*Ta*). HED is characterized by absence or hypoplasia of teeth, hair, nails, lacrimal glands, salivary glands, mammary glands, sweat glands, and sebaceous glands. The EDA protein, an *EDA* (*tabby* in mice) gene product, binds to the EDA receptor (edar). The Eda/edar signaling pathway is essential for the mesoderm-ectoderm interaction that controls the formation of ectodermal structures such as the skin, hair follicles, sweat glands, and teeth. Tabby mice SMGs are hypoplastic and exhibit smaller acini, but terminal differentiation of acinar cells is not impaired [32]. Interestingly, Edar-deficient downless (*dl*) mice SMGs are severely dysplastic, unlike tabby mice. The acini and ducts are absent in downless mice SMGs. Eda/Edar proteins localize in SMG epithelia at the site of lumen formation after the pseudoglandular stage and are essential for lumen formation and histodifferentiation of the epithelia [33].

## 3. Regeneration of the Salivary Gland in Experimental Models

 Salivary glands are well-differentiated tissues with a slow turnover time (>60 days), and damaged cells are replaced by newly generated cells. Regeneration can be either progenitor dependent or progenitor independent (autologous cell division). Normally, salivary gland cells are mainly generated from autologous cell division, and differentiated acinar cells divide to generate new ones. However, in disease/injury states caused by massive injury, autologous division does not sufficiently maintain tissue functions, and progenitor-dependent regeneration is activated. In this section, we will introduce experimental models of tissue injury and tissue stem cells isolated from salivary glands.

### 3.1. Radiation-Induced Hyposalivation

 Radiation causes cell membrane damage and induces apoptosis or loss of function in cells. In addition to acute acinar cell loss and interstitial fibrosis, vasculature dilatation occurs in irradiated salivary glands. Capillary endothelial cells are damaged and capillary permeability is increased. Therefore, capillary endothelial cells are detached from the basal lamina, and large blood vessels are abnormally dilated. Vascular damage induces secondary loss of functions of salivary glands.

 Vascular damage after irradiation can be improved by cytokine treatment. Granulocyte colony stimulating factor (G-CSF) mobilizes bone marrow cells (BMCs) to injured salivary glands by enhancing BMC recruitment. Mobilized BMCs differentiate into CD31-positive vascular endothelial cells in blood vessels to repair vascular damage and ameliorate the secretory function [34]. Radiation-induced hyposalivation could be ameliorated by pilocarpine administration before irradiation [35]. Pilocarpine enhances undamaged cell proliferation in the acini and ID compartment.

### 3.2. Duct Ligation Model

 Experimental excretory duct ligation causes severe outflow-obstruction atrophy in the salivary gland. Histopathological changes such as acinar cell loss, ductal proliferation, increased intralobular fibrous tissue, and basement membrane thickness are observed. These sequences of changes are considered a consequence of duct ligation, and this condition is associated with both degenerative and regenerative changes. The most prominent degenerative change is acinar cell depletion. High backpressure due to duct ligation presumably induces depletion of almost all acinar cells through apoptosis [36], necrosis, and autophagy [37]. High backpressure also raises intraductal pressure to dilate the intralobular duct. The emerging vacant areas after acinar cell depletion are occupied with small epithelial cells forming ductal structures. Ductal proliferation is also observed in other injured glandular organs such as the liver and the pancreas [38].

 When excretory duct obstruction occurs in rat salivary glands, the duct systems are charged with saliva containing the growth factors secreted from convoluted ductular cells. ID cells and other stem/progenitor cells are exposed to growth factors and may begin cell division if it could be responsive. In this manner, ductal proliferation is considered a regenerative change. Presumably, proliferating glandular cells participate in new acini formation in the duct-ligated salivary glands. The differentiation ability of the proliferated duct-like cells is comparable to that of ID cells [39], and these duct-like cells express SMG-B and PSP, which are normally expressed in both acinar cell precursors during development and ID cells [40]. The acini of the regenerating gland after deligation exhibit Alcian blue/PAS-double positive acinar cells that are present in embryonic salivary glands. These findings indicate that regeneration of acinar cells is similar to the cytodifferentiation process that occurs during organogenesis [40]. 

## 4. Tissue Stem/Progenitor Cells of the Salivary Gland

### 4.1. Mesenchymal Stem Cell Related

 The salivary glands contain mesenchyme cells from the embryonic period, and the adult salivary gland tissue is organized from various types of mesenchymal cells. Vascular epithelium, fibroblasts, and adipocytes are observed in interstitial tissues. These mesenchymal cells originate from mesenchymal stem cells (MSCs). In the salivary glands of aged humans, the volume of the exocrine tissue is decreased and replaced by adipose and fibrous tissues, because both acinar and duct cells lose their autologous division capabilities. Consequently, MSCs are required for salivary gland tissue.

 Stem cells with the ability to differentiate into mesenchymal cells were isolated from human parotid glands [41]. Common stem/progenitor cells were also isolated from human SMGs and the pancreas [42]; these salivary gland stem cells (SGSCs) and pancreatic stem cells (PSCs) clearly showed the ability to differentiate into all 3 mesenchymal lineages *in vitro*. Interestingly, both SGSCs and PSCs expressed nestin, an intermediate filament expressed in the neural stem cells, and they differentiated into neural marker-expressing cells. The neuronal markers protein gene product 9.5 (PGP9.5) and neurofilament (NF), and the glial marker glial fibrillary acidic protein (GFAP) are observed in these cell populations. It is unclear whether SGSCs that share MSC characteristics are multipotent, and the differences between SGSCs and MSCs need to be clarified in the future.

### 4.2. Bone-Marrow-Derived Cells

 A recent study indicated that transplantation of bone-marrow-derived cells (BMDCs) effectively rescues salivary gland functions in postirradiated mice [43]. Transplanted BMDC survival in recipient mouse salivary glands are certified by cross-sex transplantation. In this study, Y-chromosome-positive donor BMDC-derived acinar and duct cells were observed. The concentration of EGF in the saliva, the salivary flow rate, apoptotic activity, and the acinar cell area in damaged tissue were improved. BMDC transplantation is effective for hyposalivation treatment, and the salivary gland epithelium that directly differentiates from BMDCs may mainly improve functional recovery [43]. The Sca-1- and c-kit-positive BMDCs may have the ability to differentiate into the salivary gland epithelium.

### 4.3. C-Kit-Positive Duct Cells

 Mice cells expressing Sca-1, c-kit, and musashi-1 (Msi-1) have been isolated and characterized [44]. C-kit-positive cells also exist in the excretory duct of human salivary glands [45]. These c-kit-expressing cells divide to form a unique cell cluster called the “salisphere.” Salisphere-forming cells are thought to originate from duct cells because they express the duct cell markers CK7 and CK14. These salisphere-forming cells also express low levels of amylase mRNA, the expression of which is increased by 25-fold according to the increase in size of the salisphere. In contrast to amylase expression, the number of cells expressing stem cell markers is decreased in salispheres, and c-kit- and Sca-1-expressing cells exist only in the outermost layer of large salispheres (diameter, >50 *μ*m). These findings suggest that salisphere-forming cells represent salivary gland stem/progenitor cells. Cell differentiation accompanied by sphere formation was also reported in neural stem/progenitor cells. Similar to the salisphere, the neurosphere is formed by the aggregation of nestin-expressing neural stem/progenitor cells, which differentiate according to sphere growth.

### 4.4. Intercalated Duct Cells

 The ID is a small duct connecting the terminal acini and striated duct. The lumen of the ID surrounding a single layer of low small cuboidal cells with less intracellular organelles such as the rough endoplasmic reticulum and Golgi apparatus are called ID cells. In young adult female mice, 3 parenchymal cell types exist: (1) acinar, (2) ID and granular ID, and (3) granular and striated duct cells. The relative proportions of these 3 cell types are 43%, 18%, and 39%, respectively [46].

 Histological studies with [^3^H]-thymidine-labeled mice revealed that both acinar and duct cells proliferate by autologous cell division and differentiation of ID cells [46]. In the normal salivary gland, aging and/or damaged acinar and duct cells are continuously replaced by new ones. In young (<10–12 weeks) female mice, new acinar cells are mainly derived from the cytodifferentiation of ID cells. In contrast, new acinar cells in male and old female mice originate from autologous cell division [46]. These findings indicate that differentiated acinar and duct cells have the ability to divide, and that ID cell cytodifferentiation is not an exclusive mechanism by which tissue cells and functions are maintained. A [^3^H]-Thymidine labeling also showed that the ID cell compartment had a higher labeling index (LI) than acinar, striated duct, and granular duct cells. According to the time course after [^3^H]-thymidine injection, the LI of ID cells decreased, whereas the LI gradually increased in all other types of parenchymal cells. This study indicates that ID cells differentiate into both acinar and striated/granular duct cells, and that ID cells therefore play the role of progenitor cells in adult salivary glands.

### 4.5. Laminin-Producing Cells

 In the duct-ligated salivary gland, ductal proliferation occurred via the proliferation of small duct-like cells, concurrently with the appearance of unique cells producing laminin. Laminin-producing cells formed small clusters rather than duct-like structures in the interstitium of duct-ligated salivary glands in rats. These cells did not originate from proliferating small duct-like cells, because duct-like cells are not laminin positive [47].

 It is acceptable that some part of the regeneration process is similar to the developmental pathway. As described above, the immature acini that emerged in the deligated salivary gland exhibited the perinatal protein SMG-B, a marker of proacinar cells. The new acinar cells that are regenerated differentiated from the ductal cells after they underwent branching similar to embryonic glandular development [40]. Similarly, the appearance of laminin-producing cell clusters during regeneration may be an example of similarities between the regeneration process and the developmental pathway. The laminin-producing cell cluster is similar to the embryonic salivary epithelia, which express *α*6-integrin and laminin simultaneously.

 SGP cells isolated form duct-ligated rats were positive for Thy-1 (CD90), *β*1-integrin (CD29), and *α*6-integrin (CD49f) [47]. Although Thy-1 expression was common in rat, swine [48], and human SGPs [49], mouse SGPs were negative for Thy-1 but positive for Sca-1/c-kit [50]. As described above, the Sca-1 and c-kit antigens are also expressed in sphere-forming cells and BDMCs, and Sca-1/c-kit antigens may therefore be definitive salivary gland stem cell markers in mice.

**Figure 2:**
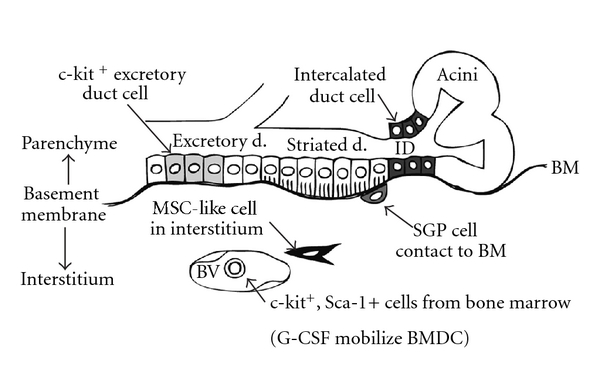
Tissue location of salivary gland stem/progenitor cells. ID cells (dark grey) and c-kit-positive excretory duct cells (light grey) are parenchymal cells that rest on the basement membrane (BM). Bone-marrow-derived cells are present in the blood vessel, and MSC-like cells (black) in the interstitium, but they are not in contact with the BM. On the other hand, SGP cells (grey) do not rest on the BM, but are in contact with the BM. Abbreviations: BM: basement membrane; BV: blood vessel; ID: intercalated duct; d.: duct.

 Human SGP cells were isolated from adult salivary glands but not from ligated glands. Human SGP cells express common SGP markers as well as p75NGFR, which serve as a location marker for SGP cells in normal salivary gland tissues [49]. It is interesting that p75NGFR is also expressed in basal/basal-like cells that are stem cells of human airway epithelium [51]. Human SGP cells located periductally come into contact with the BM but, unlike other epithelial cells, do not rest on the BM (Figure 2). 

## 5. Cytodifferentiation of Isolated Salivary Gland Stem/Progenitor Cells

### 5.1. In Vivo Study: Cell Transplantation

 Regeneration of salivary glands mainly results from autologous cell division in adult mice; therefore, the survival rate of transplanted stem/progenitor cells may be low if the recipient glands are not widely damaged. Therefore, local irradiation is an excellent model for transplantation of salivary gland stem cells. Radiation (15 Gy) induces irreversible damage in recipient mice salivary glands, and the restoration of saliva production after cell transplantation reflects acinar cell restoration by transplanted cells [44]. In this model, c-kit-positive duct cells isolated from mice salivary glands survived to differentiate into acinar cells and restored saliva production in irreversibly irradiated recipient salivary glands, indicating that c-kit-positive cells have the capacity to regenerate damaged salivary glands.

**Figure 3:**
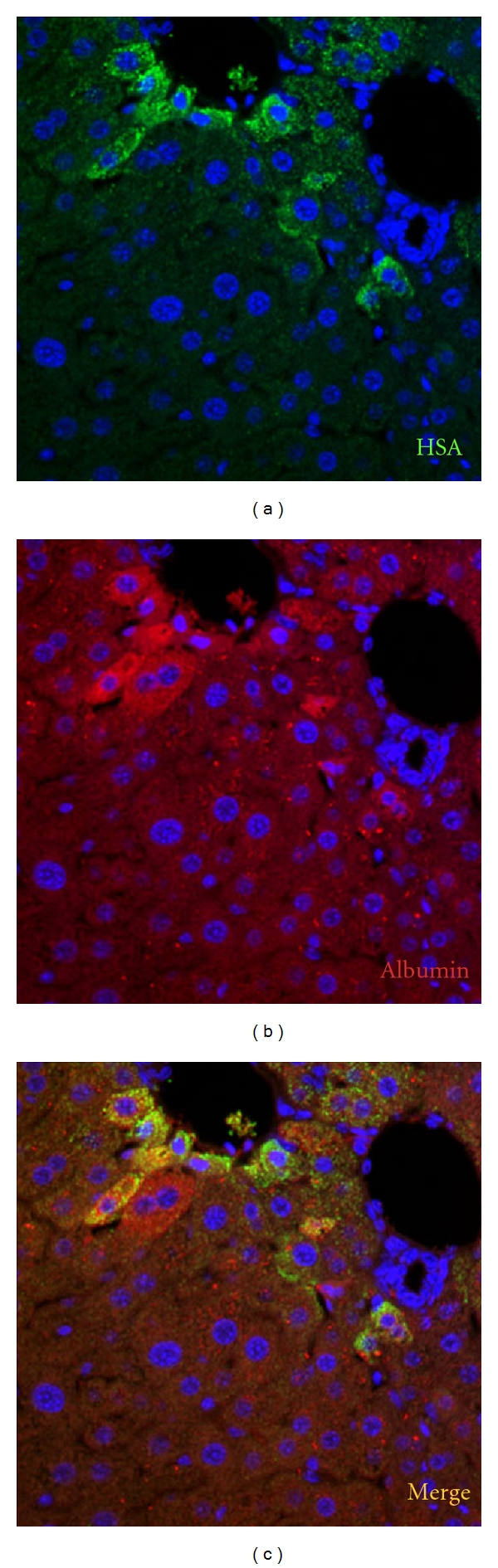
Transplantation of human SGP cells into the hepatectomized liver. RAG−/− mice (4 to 6 weeks old) served as recipients for cell transplantation into the liver. Mice were hepatectomized under anesthesia according to the methods described by Higgins and Anderson immediately before cell transplantation. Cultured human SGP cells (approximately 4.0 × 10^4^ cells/*μ*L) were suspended in Dulbecco's medium. For transplantation, a cell suspension (5 *μ*L, 2.0 × 10^5^ cells/mouse) was injected into the residual lobe (right anterior lobe) of hepatectomized recipient mice with a Hamilton syringe. Several focal necrotic lesions became apparent in the recipient liver as early as 2 days after cell transplantation. Tumor formation was not observed microscopically in the regenerating liver 4 weeks after transplantation. In order to detect cells of human origin in transplanted mouse liver specimens, we performed immunohistochemical analysis with an anti-human hepatocyte-specific antigen (HSA) antibody (Ab) (clone OCH1E5, DAKO Cytomation) and an anti-human albumin Ab (DAKO Cytomation). Clone OCH1E5 is an anti-human hepatocyte-specific antibody, and the antigen recognized by this antibody is present in normal human hepatocytes. The antibody reacts with human hepatocytes to produce a distinct, granular, cytoplasmic stain, but does not react with mouse hepatocytes. Immunofluorescence staining for HSA and human albumin in the recipient mice liver 4 weeks after human SGP cell transplantation. (a) shows HSA staining and (b) shows albumin staining. The anti-human albumin Ab weakly cross-reacted with mouse albumin. (c) is a merged image of (a) and (b). Nuclei were counterstained with DAPI. Original magnification, ×200.

 Cell transplantations into organs other than salivary glands have also been performed, and these studies demonstrate the transdifferentiation capabilities and multipotency of stem cells. Salivary progenitor cells could differentiate into both duct and acinar cells, and the capability of differentiation should be restricted. Therefore, differentiation into somatic cells other than salivary glands cells is difficult for progenitor cells. We performed SGP cell transplantation into the hepatectomized liver of a recipient animal. Both rat and mouse SGP cells survived to differentiate into hepatocyte-like cells in recipient livers [47, 50]. Similar to rat and mouse SGP cells, human SGP cells also differentiated into albumin-producing cells when transplanted into the hepatectomized liver (Figure 3), suggesting that human SGPs have the ability to differentiate into hepatic-type cells. Transplanted human SGP cells differentiated to express albumin and the HSA antigen, which is only expressed in differentiated human hepatocytes. Thus, the multipotency of SGP cells was demonstrated by successful hepatic transdifferentiation.

### 5.2. In Vitro Study

 MSC-related cells isolated from salivary glands differentiated into adipogenic, chondrogenic, and osteogenic lineages *in vitro* [41, 42]. According to Pittenger and colleagues, these cells can be induced to differentiate into mesoderm-derived lineages in cell culture containing added supplements and growth factors [52].

 Salivary stem/progenitor cell cytodifferentiation also depends on cell aggregation. C-kit-positive duct cells isolated from human salivary glands form a sphere-like structure called a salisphere. During salisphere formation, c-kit-positive cells differentiate into amylase-producing acinar cells *in vitro*. Sphere formation accelerates the cytodifferentiation of c-kit-positive cells. In SGP cells, cell clusters of various sizes were formed according to the culture period. Each SGP cell synthesizes to retain intracellular laminin, however, SGP cells forming clusters lose intracellular-laminin and clusters are surrounding laminin [47]. These findings suggest that the laminin secreted by SGP cells facilitates the formation of cell clusters/aggregates in culture. Laminin synthesis/secretion is also observed in the salivary epithelium during BrM, as described above. SGP cell clusters contained differentiated SGP cells that produced albumin (hepatocyte-like) or insulin/glucagon (pancreatic endocrine-like) [47]. Thus, cluster formation is essential for SGP cell cytodifferentiation.

### 5.3. Morphogenesis Driven by Stem Cells

 Many kinds of stem/progenitor cells have been isolated from adult salivary glands, as listed above. These cells were characterized by expression of stem cell markers, formation of sphere/clusters, differentiation into mesenchymal or amylase-producing cells, and tissue location. Among them, what is the best hallmark of stem/progenitor cells? In addition, what is the most important stem cell characteristic?

 Classically, a stem cell is capable of both unlimited self-renewal and multipotency. The regeneration of damaged tissue is one of the most important capabilities of stem/progenitor cells, especially in clinical medicine. Progenitor cells that rapidly divide but exhibit restricted differentiation potential may be more useful than tissue stem cells for regeneration of damaged tissues. In contrast, a stem cell has the capability to restructure a whole tissue/organ when located in the appropriate environment. For instance, completely dissociated primary embryonic SMG epithelial cells self-organized into structures and underwent BrM when grown in Matrigel [53]. In a similar fashion, isolated stem cells are expected to form a branching organ-like structure [54]. Self-organization is essential for morphogenesis, and this characteristic should therefore be a prerequisite for stem cells of solid tissue. Until now, the capability of salivary stem/progenitor cells to self-organize has not been developed or clearly reported.

**Figure 4:**
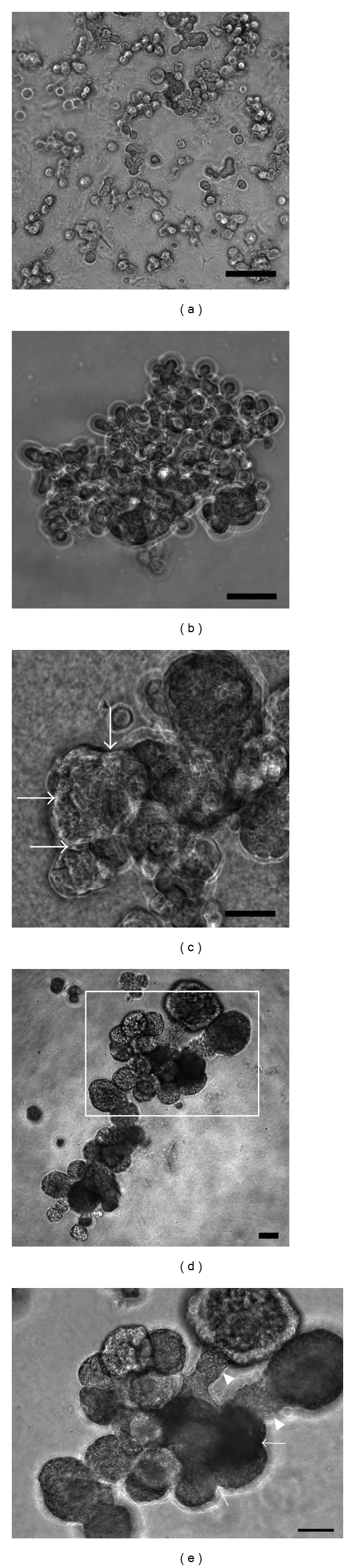
Organization of human SGP cells into a branching organ-like structure. A total of 3,000 cultured human SGP cells were suspended in 100 *μ*l of differentiation medium and seeded over a thick gel layer in each well of a 96-well plate. The seeded cells were cultured on 2 types of gels: Matrigel and PuraMatrix Peptide Hydrogel (BD Biosciences). Culture media were changed at 3-day intervals in both cell cultures. Matrigel contains both ECM proteins and several growth factors, whereas PuraMatrix peptide hydrogel consists of standard amino acids (1% w/v) and 99% water. The differentiation medium consists of Williams' E medium supplemented with 1× ITS-X (GIBCO Invitrogen) and 20 ng/mL of recombinant human EGF (Sigma). (a) shows seeded cells on each gel at day 0. (b) After 7 days, human SGP cells cultured on PuraMatrix failed to form a branching structure and exhibited only loose cell-to-cell aggregation without a round end-bud and stalk. (c), (d), (e) In comparison, human SGP cells cultured on Matrigel organized into a branching structure with stalks and round end buds. (c) shows human SGP cells cultured on Matrigel for 7 days, and (d) shows the culture for 14 days. (e) is a magnified image within the frame of (d). The arrowhead indicates the stalk portion without the lumen in (e). Arrows indicate cleft-like portions in the round end buds in panels (c) and (e). Original magnifications, ×40 (d), ×100 (a, b, c, e). Scale bars =100 *μ*m.

 In order to organize branching structures, we cultured human SGP cells on Matrigel containing exogenous EGF, as reported previously [3, 53]. Human SGP cells cultured on Matrigel divide to organize branching structures with stalk and end buds; on the other hand, they fail to organize into branching structures when cultured on Puragel containing no ECM proteins (Figure 4). These results revealed that human SGP cells have self-organizing ability, and Matrigel with exogenous EGF, which is required for BrM of salivary epithelium *in vitro*, is also necessary for self-organization of stem cells. The appearance of this branching structure is similar to salivary rudiments of the pseudoglandular stage that are characterized by few large round end buds with a short thick stalk; however, it was not possible to identify the proximal (stalk) end. It is thought that the characteristics of human SGP cells are similar to those of the salivary epithelium of the pseudoglandular stage, which expresses laminin and *α*6-integrin simultaneously; it is therefore thought that human SGP cells are capable of self-organization into a branching structure. However, this culture system could not induce further development into this branching structure.

 In conclusion, we established a sequential procedure for organization of a branching structure, similar to the salivary rudiment of the pseudoglandular stage, using tissue stem cells from adult human salivary glands. Further investigations are required for induction of terminal differentiation of this branching structure.

## Figures and Tables

**Table 1:** Acinar cell type of the major salivary glands.

	Submandibular^1^	Sublingual	Parotid	Ref.
Human	Predominantly serous (~90%)	Purely mucous	Purely serous	[4, 5]
Mouse	Seromucous	Mainly mucous	Purely serous	[6]
Rat	Seromucous	Mainly mucous (>90%)	Purely serous	[6–9]

^1^The mucous cells in the submandibular glands of mice and rats exhibit sexual dimorphisms. For example, the percentage of mucous cells in SMG is 57.1% at 1 month and reaches 100% at 6 months in male rats, whereas that in the female rats is 60.0% and 28.5%, respectively. The frequency of mucous cells in SMG can be influenced by androgens [9].

In contrast, mucous acinar cell volumes of the human SMG comprise approximately 5–10% of total acinar cells. The sex- and age-related differences in mucous acinar cell volume are not significant in humans [5].

## Abbreviations

BM: Basement membrane
BMC: Bone-marrow cell
BMDC: Bone-marrow-derived cell
BrM: Branching morphogenesis
ECM: Extracellular matrix
EDA: Ectodysplasin-A
EDAR: Ectodysplasin-A receptor
EGF: Epidermal growth factor
GCT: Granular convoluted tubule
HED: Hypohydrotic ectodermal dysplasia
ID: Intercalated duct
MSC: Mesenchymal stem cell
NGFR: Nerve growth factor receptor
PAS: Periodic acid-Schiff
PSC: Pancreatic stem cell
PSP: Parotid secretory protein
SGP: Salivary gland-derived progenitor
SGSC: Salivary gland stem cell
SMG: Submandibular gland
SMG-B: Submandibular gland protein B
TGF: Transforming growth factor.
